# Antimicrobial susceptibility and neonatal sepsis in a tertiary care facility in Nigeria: a changing trend?

**DOI:** 10.1093/jacamr/dlac100

**Published:** 2022-09-30

**Authors:** Nkoyo O Uwe, Beatrice N Ezenwa, Iretiola B Fajolu, Philip Oshun, Stella T Chukwuma, Veronica C Ezeaka

**Affiliations:** Department of Paediatrics, Lagos University Teaching Hospital (LUTH), Lagos, Nigeria; Department of Paediatrics, Lagos University Teaching Hospital (LUTH), Lagos, Nigeria; Department of Paediatrics, College of Medicine University of Lagos, Lagos, Nigeria; Department of Paediatrics, Lagos University Teaching Hospital (LUTH), Lagos, Nigeria; Department of Paediatrics, College of Medicine University of Lagos, Lagos, Nigeria; Department of Medical Microbiology, Lagos University Teaching Hospital (LUTH), Lagos, Nigeria; Department of Medical Microbiology, College of Medicine University of Lagos, Lagos, Nigeria; Department of Medical Microbiology, Lagos University Teaching Hospital (LUTH), Lagos, Nigeria; Department of Paediatrics, Lagos University Teaching Hospital (LUTH), Lagos, Nigeria; Department of Paediatrics, College of Medicine University of Lagos, Lagos, Nigeria

## Abstract

**Background:**

Neonatal sepsis remains one of the leading causes of morbidity and mortality in neonates, especially in developing countries.

**Objectives:**

To determine the prevalence, common bacterial pathogens, and the antibiotic susceptibility pattern of neonatal sepsis at the Lagos University Teaching Hospital (LUTH), Lagos, Nigeria.

**Methods:**

This was a cross-sectional study of neonates who presented at the facility with symptoms and signs of sepsis from January 2017 to October 2017. Demographic and clinical data were extracted using a structured questionnaire. Blood culture, urine and CSF were collected and cultured on blood and MacConkey agar. Bacterial isolates were identified using Microbact 24E system and biochemical tests. Antibacterial susceptibility testing was done using the modified Kirby–Bauer disc diffusion method.

**Results:**

Two hundred and ninety neonates were recruited during the study period. Seventy-three (25.2%) neonates had culture-proven sepsis. One (0.3%) neonate had meningitis and no neonates (0%) had confirmed urinary tract infection. Of the 73 neonates with positive blood cultures, 56 (76.7%) had early-onset sepsis and 17 (23.3%) had late-onset sepsis. Gram-negative bacilli accounted for 60.3% of all isolates. Predominantly isolated pathogens were *Staphylococcus aureus* (20.5%), CoNS (19.2%) and *Klebsiella pneumoniae* (13.7%). The isolates were most susceptible to levofloxacin and amikacin.

**Conclusions:**

Neonatal sepsis is still a huge burden in the newborn. *S. aureus*, CoNS and *K. pneumoniae* are the prevalent pathogens in the local facility, with good susceptibility to levofloxacin and amikacin. Maintaining regular antibiotic surveillance for appropriate empirical antibiotics is important as part of neonatal care.

## Introduction

Neonatal sepsis is defined as a clinical syndrome characterized by systemic signs and symptoms of infection with bacteraemia in the first 28 days of life.^1^ It is a medical emergency and requires urgent diagnosis and treatment to prevent mortality.^2^ Globally, it is estimated that annually 3 million newborns suffer from sepsis and that 30% of deaths in under-fives are due to neonatal sepsis.^3^ Worldwide, the incidence of culture-proven sepsis ranges from 0.9/1000–1.5/1000 live births in developed countries to 16/1000 live births in developing countries.^4^ In Nigeria, hospital-based studies documented the prevalence of neonatal sepsis at 7.04/1000–22.9/1000 live births but it accounts for 31.5% of total neonatal deaths.^5,6^

Neonates are most susceptible to infectious agents due to the immaturity of their immune system. In neonates the systemic inflammatory response to infection is reduced due to low immunoglobulin levels, low complement activity, low neutrophil storage and impaired capacity of the neutrophils to migrate from the blood to sites of infection.^7^

Evaluating a neonate with suspected sepsis will include culture-based diagnosis with direct isolation of pathogens from blood, CSF, urine and swabs of discharges from various lesions obtained aseptically, and an indirect sepsis screening test. Culture-based diagnostic investigations for suspected sepsis establish the presence or absence of neonatal sepsis.^8^ The isolation of an organism from a culture-based test and its antibiotic susceptibility is an important component of managing sepsis.

The microbial aetiology of neonatal sepsis and the antibiotic susceptibility pattern is variable from one geographical region to another and changes from time to time. The causative organisms of neonatal sepsis have shown increasing resistance to the commonly used empirical antimicrobial agents.^9^ This may be due to unnecessary overuse of these antibiotics over a long time, thus making treatment difficult.^9^ It is imperative, therefore, that the local aetiology of neonatal sepsis and the antibiotic susceptibility of any neonatal unit be regularly reviewed and updated to determine the empirical antibiotics that can be used in the initial treatment before a definitive diagnosis is made.

In the neonatal unit of our hospital, two previous studies looked at causative organisms of neonatal sepsis 22^10^ and 8 years^11^ ago, respectively, prior to this study. Both studies only looked at blood pathogens and may have missed neonates with meningitis or urinary tract infection (UTI). The present study, therefore, aimed to determine the current pattern of bacterial organisms in our neonatal unit from blood, CSF and urine and their antibiotic susceptibility patterns.

## Materials and methods

### Study location

This study was conducted in the neonatal unit of the Lagos University Teaching Hospital (LUTH), Lagos State, Nigeria. The hospital is one of the largest tertiary healthcare hospitals in Nigeria with a 760 bed capacity and serves as a referral hospital for patients from private hospitals and general hospitals in and out of Lagos State. The neonatal unit has 37 cots and 41 incubators and serves the immediate community and its environs.

### Study design

This was an observational, cross-sectional study of inborn and outborn neonates aged 0–28 days who were admitted to our hospital with risk factors, symptoms and signs suggestive of sepsis from January 2017 to October 2017.

### Study population

Using data from a previous study in our environment, a sample size of 290 participants was calculated, and consecutive neonates, both term and preterm, aged 0–28 days, presenting with risk factors and/or clinical features suggestive of sepsis and admitted into the neonatal unit were recruited for the study.

The inclusion criteria were presence of risk factors for sepsis such as being preterm, history of foul-smelling amniotic fluid, prolonged rupture of membranes greater than 18 h, peripartum pyrexia in mother, chorioamnionitis, home delivery and prolonged resuscitative efforts greater than 10 min in the baby. In addition, included babies presented with clinical features of suspected sepsis such as fever, hypothermia, poor suck, seizures, abdominal distension, respiratory difficulties, lethargy or jaundice.

Neonates were excluded if they did not meet inclusion criteria, were exposed to antibiotics use prior to hospital admission or their mothers were already on antibiotics at the time of birth. This was to reduce resistance of bacterial isolates to tested antibiotics.

### Ethics

Ethical approval for the study was obtained from the Lagos University Teaching Hospital Health Research Ethics Committee. Parental consent was obtained for all infants before recruitment.

### Data collection

Socio-demographic characteristics and details of perinatal events were obtained from patients’ relatives/carers using a structured questionnaire designed for this study. Parental educational level and occupation were used to determine the socio-economic class of each participant using the Socio-economic class (SEC) classification system by Oyedeji.^12^ Scores of 1 to 5 were given for each criterion in the SEC classification system, with 1 being the highest and 5 the lowest. The scores were added for both parents, and the average (approximated to whole numbers) gave the SEC. SEC 1 and 2 were regarded as the upper class, SEC 3 as middle class and SEC 4 and 5 as lower classes.

### Sample collection and processing

Blood, CSF and urine samples were collected from each neonate within 1 h of admission following standard procedures by the most senior paediatric registrar on duty who was previously trained on the aseptic technique of blood culture sampling. Two millilitres of blood were withdrawn from the venepuncture site aseptically, applying universal precautions. The blood drawn from each neonate was aseptically dispensed into the BD BACTEC™ Peds Plus/F (BD, USA) aerobic culture bottle (one bottle per neonate). The culture bottles were incubated in the BD BACTEC™ 9050 instrument for a maximum of 5 days if no bottle flagged positive earlier than the fifth day. CSF and urine samples were also obtained aseptically into different sterile universal bottles.

Urine, CSF and positive blood culture samples had a Gram stain before subculture on appropriate media including 5% sheep blood agar for Gram-positive cocci (GPC) and MacConkey agar for Gram-negative bacilli (GNB). Urine samples were analysed for epithelial cells, pus cells and casts, while CSF samples were also analysed for cell count, protein and glucose estimation.

Isolates were identified by biochemical tests including Microbact 24E (Oxoid Ltd, Hampshire, UK) for GNB, and catalase and coagulase tests for GPC.

### Antimicrobial susceptibility testing

Antibiotic susceptibility testing was done using the modified Kirby–Bauer disc diffusion method with Mueller–Hinton agar (Oxoid Ltd, Hampshire, UK) following guidelines provided by CLSI.^13^ The antibiotics used for susceptibility testing were based on commonly used antibiotics in the environment and included, for GPC: amikacin 30 µg, clindamycin 2 µg, cefoxitin 30 µg, levofloxacin 5 µg and vancomycin for MRSA and CoNS; for GNB: amikacin 30 µg, cefotaxime 30 µg, cefepime 30 µg, levofloxacin 5 µg, piperacillin/tazobactam (100/10 µg) and meropenem 10 µg. Antimicrobial susceptibility results were interpreted as susceptible or resistant based on the interpretative criteria of the 26^th^ edition of CLSI’s M100.^13^ The intermediate susceptibility was included under the susceptible group in this study due to the small numbers and for ease of analysis. A cefoxitin disc was used to detect MRSA.

### Data analysis

Clinical and laboratory data was entered into a Microsoft Excel 2013 spreadsheet. Data were then exported and analysed using the International Business Machine (IBM) Statistical Package for Social Sciences (SPSS) for Windows version 23. Frequency tables were generated for categorical variables such as sex, mode of delivery, risk factors, clinical features, organisms isolated and susceptibility pattern. Continuous variables were presented as means and SDs for normally distributed data and as median and IQR for skewed data. Charts were used for data presentation where appropriate. Test of association between categorical data was done using the chi-squared (χ²) test or Fisher’s exact test where expected values in any of the cells were less than five. A probability value less than 0.05 (*P* value < 0.05) was considered as statistically significant at 95% CI.

## Results

The total number of admissions in the neonatal unit during the study period was 596 neonates. Two hundred and ninety neonates who met the inclusion criteria were enrolled into the study. Of these, 150 (51.7%) were male, with a male:female ratio of 1.1:1. The gestational age of the neonates ranged from 25 to 42 weeks with a mean of 35.4 ± 4.4 weeks. One hundred and forty-six (50.30%) were preterm neonates. Two hundred and twenty-five neonates (77.6%) presented in the first 72 h of life. There were more outborn neonates (202, 69.7%) with suspected sepsis compared with inborn neonates (88; 30.3%). Table 1 shows the baseline characteristics of the study participants.

**Table 1.** Socio-demographic characteristics of study population

Variables	Frequency (*N* = 290)	Percentage (%)
Gender		
Male	150	51.7
Female	140	48.3
Gestational age (weeks)		
Preterm (<37)	146	50.3
Term (37–42)	144	49.7
Post term (>42)	0.0	0.0
Mean ± SD	35.4 ± 4.4	
Mode of delivery		
Vaginal	183	63.1
Caesarean section	107	36.9
Onset of illness (h)		
Early (≤72)	225	77.6
Late (>72)	65	22.4
Median ^a^(Q1– Q3)	23.0 (19.0–72.0)	
Place of birth		
Inborn	88	30.3
Outborn	202	69.7
Weight on admission (kg)		
< 2.5	172	59.3
≥ 2.5	118	40.7
Mean ± SD	2.2 ± 0.9	
Social class of parents		
Low	141	48.6
Middle	94	32.4
High	55	19.0

a Q1, first quartile; Q3, third quartile.

Seventy-three (25.2%) participants had positive blood cultures. One (0.3%) neonate had a positive CSF culture and a corresponding positive blood culture, thus giving the prevalence of confirmed neonatal meningitis in this study as 0.3%. There were no positive urine cultures.

Table 2 shows the bacterial isolates from positive blood cultures. GNB accounted for the majority of the isolates (44; 60.3%). Major pathogens from blood were *Staphylococcus aureus* (15; 20.5%), CoNS (14; 19.2%) and *Klebsiella pneumoniae* (10; 13.7%).

**Table 2.** Bacterial isolates from positive blood cultures

Isolates	Frequency	Percentage (%)
GPC		
*S. aureus*	15	20.5
CoNS	14	19.2
		
GNB		
*K. pneumoniae*	10	13.7
*Burkholderia cepacia*	5	6.8
*P. aeruginosa*	5	6.8
*Escherichia coli*	4	5.5
*Acinetobacter baumannii*	3	4.1
*Pseudomonas fluorescens*	3	4.1
*Klebsiella oxytoca*	3	4.1
*Klebsiella ozaenae*	2	2.7
*Citrobacter freundii*	2	2.7
*Acinetobacter lwoffii*	1	1.4
*Alcaligenes faecalis*	1	1.4
*Pseudomonas stutzeri*	1	1.4
*Morganella morganii*	1	1.4
*Raoultella ornithinolytica*	1	1.4
*Serratia marcescens*	1	1.4
*Serratia rubidaea*	1	1.4
Total	73	100

*K. pneumoniae* (100%) was responsible for the only case of meningitis. The patient with meningitis had the same organism isolated from blood. Table 3 shows the distribution of blood culture isolates by gestational age, place of birth and type of sepsis. *S. aureus* (18.6%), *K. pneumoniae* (16.3%) and *Pseudomonas aeruginosa* (11.6%) were the major isolates among preterm neonates. *S. aureus* (37.5%) and CoNS (17.5%) were the major causes of Gram-positive sepsis among the inborn and outborn patients respectively. *K. pneumoniae* was the commonest cause of Gram-negative sepsis in the unit. *S. aureus* and CoNS were the predominant organisms isolated in both early-onset sepsis(EOS) and late-onset sepsis (LOS).

**Table 3.** Blood culture isolates by gestational age, place of birth and type of sepsis

Isolates	Gestational age		Place of birth		Type of sepsis	
	Preterm, *n* (%)	Term, *n* (%)	Inborn, *n* (%)	Outborn, *n* (%)	EOS, *n* (%)	LOS, *n* (%)
Gram positive						
*S. aureus*	8 (18.6)	7 (23.3)	6 (37.5)	9 (15.8)	12 (21.4)	3 (17.7)
CoNS	3 (7.0)	11 (36.7)	4 (25.0)	10 (17.5)	11 (19.6)	3 (17.7)
Gram negative						
*K. pneumoniae*	7 (16.3)	3 (10.0)	2 (12.5)	8 (14.0)	8 (14.3)	2 (11.8)
*P. aeruginosa*	5 (11.6)	0 (0.0)	1 (6.2)	4 (7.0)	4 (7.1)	1 (5.9)
*B. cepacia*	3 (7.0)	2 (6.7)	0 (0.0)	5 (8.8)	4 (7.1)	1 (5.9)
*E. coli*	1 (2.3)	3 (10.0)	0 (0.0)	4 (7.0)	3 (5.4)	1 (5.9)
*K. oxytoca*	3 (7.0)	0 (0.0)	0 (0.0)	3 (5.3)	3 (5.4)	0 (0.0)
*A. baumannii*	3 (7.0)	0 (0.0)	1 (6.2)	2 (3.5)	3 (5.4)	0 (0.0)
Other Gram-negative isolates	10 (23.3)	4 (13.3)	2 (12.5)	12 (21.1)	7 (12.5)	2 (11.8)
Total	43	30	16	57	56	17

Table 4 shows the association between types of isolate and gestational age, place of delivery, and occurrence of sepsis. Gram-positive isolates were more common among term neonates while Gram-negative isolates were more common among the preterm neonates. Gram-positive sepsis was predominant among inborn neonates while Gram-negative sepsis was more common among the outborn neonates.

**Table 4.** Association between type of isolate and gestational age, place of birth, and type of sepsis

	Gram positive, *n* (%)	Gram negative, *n* (%)	χ²	*P* value
Gestational age				
Preterm	11 (37.9)	32 (72.7)	8.743	0.003*
Term	18 (62.1)	12 (27.3)		
Place of birth				
Inborn	10 (34.5)	6 (13.6)	4.439	0.035*
Outborn	19 (65.5)	38 (86.4)		
Type of sepsis				
EOS	23 (78.3)	33 (75.0)	0.181	0.669
LOS	6 (20.7)	11 (25.0)		

*mean ‘statistically significant *P* value.’

In Table 5, the general antibacterial susceptibility pattern of isolated organisms was described. Overall, the organisms were most susceptible to levofloxacin (82.2%) and amikacin (72.6%). Gram-positive pathogens were most susceptible to vancomycin (100%) and most resistant to ampicillin/sulbactam (62.1%). The antibiotics to which most Gram-negative pathogens were susceptible were levofloxacin (86.4%) and piperacillin/tazobactam (84.1%), while they were most resistant to cefotaxime (50%).

**Table 5.** Antibacterial susceptibility pattern of blood isolates

Antibiotics	Susceptible, *n* (%)	Resistant, *n* (%)
Gram-positive isolates		
Amikacin	22 (75.9)	7 (24.1)
Levofloxacin	22 (75.9)	7 (24.1)
Ampicillin/sulbactam	11 (37.9)	18 (62.1)
Cefoxitin	13 (44.8)	16 (55.2)
Clindamycin	15 (51.7)	14 (48.3)
Vancomycin^a^ (*n* = 21)	21 (100.0)	0 (0.0)
Gram-negative isolates		
Amikacin	31 (70.5)	13 (29.5)
Levofloxacin	38 (86.4)	6 (13.6)
Cefepime	31 (70.5)	13 (29.5)
Cefotaxime	22 (50.0)	22 (50.0)
Meropenem	30 (68.2)	14 (31.8)
Piperacillin/tazobactam	37 (84.1)	7 (15.9)

a Only tested against MRSA and CoNS.

Methicillin resistance was seen in almost half (47%) of *S. aureus*. MRSA were susceptible to vancomycin and amikacin at 100% and 71.4%, respectively. CoNS were 100% susceptible to vancomycin and least susceptible to ampicillin/sulbactam at 21.4%. These are shown in Table 6.

**Table 6.** Antibacterial susceptibility pattern of GPC

Isolates	Antibiotics					
	Amikacin, *n* (%)	Levofloxacin, *n* (%)	Ampicillin/sulbactam, *n* (%)	Cefoxitin, *n* (%)	Clindamycin, *n* (%)	Vancomycin, *n* (%)
CoNS (14)	7 (50.0)	11 (78.6)	3 (21.4)	7 (50.0)	5 (35.7)	14 (100.0)
*S. aureus* (8)	5 (62.5)	6 (75.0)	6 (75.0)	8 (100.0)	6 (75.0)	NA
MRSA (7)	5 (71.4)	4 (57.1)	NA	0 (0.0)	3 (42.9)	7 (100.0)

NA, not available.

## Discussion

Our study set out to determine the current prevalence, common bacterial pathogens and the antibiotic susceptibility pattern of neonatal sepsis in our facility. We documented a prevalence of 25.2% for positive blood cultures among neonates with clinical features of neonatal sepsis. *S. aureus* was the commonest organism encountered in our patients, with levofloxacin being the commonest antibiotic the isolated organisms were susceptible to.

The prevalence of confirmed neonatal sepsis documented in this study was lower than the 34% and 35% reported in the study centre 8 and 22 years ago, respectively.^10,11^ It is also lower than the 43.5% reported elsewhere in Nigeria but higher than what was reported in Ghana, another sub-Saharan African country.^14^ When compared with what was documented for some high-income countries such as the USA (0.1%) and Poland (4%),^15,16^ it is a huge number. The implication is that though culture-positive neonatal sepsis seems to be reducing in this study, it is still a huge burden. The hospital antibiotic policy and stewardship, which has seen a new zest in the last few years, should be intensified. Infection control and prevention should be every healthworker’s responsibility. The majority of the septic babies in this study were in the outborn unit. This emphasizes the need for improved quality of care and standard of hygiene, even within communities. The reduction in the prevalence rate could also reflect increased health awareness by mothers and caregivers with improved hygiene, and improved antibiotic stewardship and other healthcare delivery services over time in the hospital. Poor infection control practices, poor standard of living and unhygienic environments play a critical role in neonatal sepsis.^17^

The prevalence of neonatal meningitis in this study is low and it is comparable to other studies elsewhere.^18,19^ Only one neonate had confirmed meningitis with CSF that grew *K. pneumoniae*. The majority of our participants were recruited within 72 h of birth and previous studies had documented very low prevalence of meningitis in EOS.^18,19^ However, a high prevalence of 17.9% was observed by Laving *et al*.^20^ in Kenya where bacteria in CSF were identified through standard culture techniques and an added advantage of a latex agglutination assay, which may have contributed to the high prevalence. This study utilized only standard aerobic culture methods of bacterial isolation.

A predominance of GNB in this study is similar to previous findings in the study centre ,where GNB accounted for 61.1%^11^ and 73%^10^ of bacteria isolated. It is also similar to reports of 78.9% in Abeokuta,^5^ Nigeria and 49.2% in South Africa.^21^ The predominance of GNB in the present study may be due to more neonates presenting with EOS as GNB is the commonest cause of infection in EOS, which the babies acquired from their mothers. Other studies elsewhere in Nigeria^22^ and India^23^ have reported a predominance of GPC over GNB. Pius *et al*.^24^ in Maiduguri, Nigeria reported equal isolation rate sfor GNB (50%) and GPC (50%). This strengthens the need for regular surveillance of bacterial pathogens in each centre as the clinical implication of the differences in the bacterial spectrum from different neonatal units will determine the empirical antibiotic choice for each neonatal unit pending retrieval of the results of the antibiotic susceptibility pattern of the organisms. Also, adequate access to blood culture and utilization of blood culture diagnostics in each facility will ensure that irrational antibiotic use is curtailed. With increased diagnostic capacity and improved use of quality assurance in facilities, the tendency to over-diagnose and place all newborns on antibiotics will be reduced and antibiotic stewardship improved. The net effect will be the desired reduction in antimicrobial resistance to the available antimicrobials in our environment.

We discovered *S. aureus* and CoNS to be the predominant organisms in the present study. This contrasts with the previous studies in the study centre in 1995 and 2011 where *K. pneumoniae* was the predominant organism.^10,11^ Thus, after over two decades the predominant organisms causing neonatal sepsis in our centre have changed from a predominance of Gram-negative to Gram-positive organisms. This is remarkable and has implications for patient care. The finding of *S. aureus* and CoNS as the predominant causative organisms of neonatal sepsis has also been reported by other researchers within and outside of Nigeria.^25–27^ Medugu *et al*.^22^ in Abuja, Nigeria reported a shift in predominant organisms at their study centre over one decade from *K. pneumoniae* to *S. aureus*.

*S. aureus* and CoNS were the major causative organisms in both EOS and LOS in this study. This is similar to reports by Akindolire *et al.*^28^ in Ibadan, Nigeria, who reported *S. aureus* as the major organism causing both EOS and LOS, and Jatsho *et al.*^29^ in Bhutan who reported CoNS as the major organism in both EOS and LOS. Our finding from this study is at variance with previous reports from the study centre where *K. pneumoniae* was the major organism causing both EOS and LOS.^10,11^ Our finding shows a change in pattern in the predominant cause of neonatal sepsis at the study centre within two decades from a Gram-negative organism to a Gram-positive organism and confirms that organisms causing neonatal sepsis change from time to time within the same environment. The reason for the change in causative organisms of EOS between the previous studies in the study centre and this study could be a change in the organisms colonizing the anogenital region of mothers from *K. pneumoniae* to *S. aureus*. Again, the majority of these babies were from the outborn unit, having been handled by various people from healthcare staff to family members who may be carriers of these organisms.^30^

All the isolates from blood in this study were most frequently susceptible to amikacin and levofloxacin. The Gram-positive pathogens, as a group, were more susceptible to vancomycin, amikacin and levofloxacin, with least susceptibility to ampicillin/sulbactam. This is comparable to studies by Peterside *et al.*^31^ in Bayelsa, Nigeria with susceptibility of 72%–92.9% for GPC to quinolones, and Quazi *et al.*^32^ in Bangladesh with 100% susceptibility of GPC to amikacin and 83% to vancomycin. The resistance of GPC to ampicillin/sulbactam in this study is also similar to reports from within and outside Nigeria.^33,34^ Similar susceptibility of GNB to piperacillin/tazobactam has also been reported by Kamble *et al.*^35^ at 80%–100%. Piperacillin/tazobactam and vancomycin are not readily used in sick neonates in the study centre and are reserved for both confirmed and unconfirmed cases of neonatal sepsis in which there is no clinical improvement after use of empirical antibiotics and/or second-line antibiotics. This may explain why most tested isolates were more susceptible to these drugs due to the rarity of their use as they are last-resort antibiotics and the high cost of these drugs in Nigeria also limits their use. The resistance of GNB to cefotaxime in this study, which is a first-line antibiotic in the study centre, is in conformity with other studies that have shown increasing resistance of bacteria to third-generation cephalosporins, probably due to its overuse.^32,36,37^

The susceptibility of MSSA to fluoroquinolone went from 100% in the previous study to 75% in the present study, while amikacin improved from 50% to 62.5% in its susceptibility.^10^ It is reassuring to see that MSSA was frequently susceptible to ampicillin/sulbactam (75%) in this study. Following the report of the 100% resistance in the previous studies in the centre, ampicillin was no longer used in our newborn units. The addition of a β-lactamase inhibitor (sulbactam) to ampicillin enhances the effect of ampicillin and may be responsible for its susceptibility rate in this study. Again, we demonstrated an increased susceptibility of *S. aureus* to quinolones, which has also been reported by other studies.^38,39^ The clinical implication is that infection with β-lactamase-producing strains of MSSA can be treated with less costly and more easily available drugs. However, the majority of the *S. aureus* isolates in the present study were cefoxitin resistant (methicillin resistant). There was 100% susceptibility of CoNS and MRSA to vancomycin in this study, which is similar to reports from Egypt^30^ and Iran^36^ Akindolire *et al.*^28^ in Nigeria reported high resistance of MRSA to vancomycin. Vancomycin was not tested against CoNS in the previous study at the study centre. With the limited choice of antibiotics available in our environment, the high susceptibility of MRSA to vancomycin in this study is reassuring as vancomycin is the drug of choice for MRSA to reduce the high morbidity and mortality associated with MRSA infection.

## Limitations

It is possible that some of the organisms isolated may have represented contaminants as opposed to true pathogens. For instance, the CoNS isolated may be contaminants as no molecular sub-analysis was carried out and only one set of blood cultures was carried out for each participant. However, considering the clinical presentation and the response to antibiogram tests, the possibility that the isolates may be true pathogens cannot be discounted.^40^ Strict asepsis in sample collection is routinely observed in the facility and the microbiology unit of the hospital performs regular quality assurance training and surveillance in the newborn wards. Additionally, the use of only aerobic culture media could have missed fastidious organisms since they were not specifically looked for using special anaerobic culture media.

### Conclusions

The prevalence of bacteraemia with positive blood cultures in children presenting with neonatal sepsis is still very high. *S. aureus*, CoNS and *K. pneumoniae* are the prevalent pathogens in the local facility with high susceptibility to levofloxacin and amikacin. The majority of the *S. aureus* isolates were methicillin resistant but susceptible to vancomycin. We recommend the use of levofloxacin and amikacin as the empirical antibiotic of choice in the unit while vancomycin should be reserved for severe or MDR *S. aureus* infections. Regular surveillance of local antimicrobial resistance and review of antibiotic guidelines in the neonatal unit should be maintained.
